# Magnesium Homeostasis and Magnesium Transporters in Human Health

**DOI:** 10.3390/nu17050920

**Published:** 2025-03-06

**Authors:** Man Liu, Samuel C. Dudley

**Affiliations:** Cardiovascular Division, Department of Medicine, The Lillehei Heart Institute, University of Minnesota at Twin Cities, Minneapolis, MN 55455, USA; sdudley@umn.edu

## 1. Introduction

Magnesium (Mg^2+^) used to be considered only as a passive cation associated with ATP, but this special issue reinforces the idea that Mg^2+^ has many more roles. Mg^2+^, as the second most abundant intracellular divalent cation, is important for mitochondrial function and electron transport, ATP production, macrophage activation, cell signaling, antioxidant behavior, transcriptional regulation, DNA/RNA synthesis, enzyme/kinase activation, ion channels, and many other cellular functions and activities [1,2,3,4,5,6,7].

Therefore, it is important to maintain Mg^2+^ homeostasis, stable concentrations of Mg^2+^ in the cells and the whole body. Cellular Mg^2+^ homeostasis is maintained by Mg^2+^ transporters on the cell membrane and organelles’ membranes, while the systemic Mg^2+^ homeostasis is regulated by Mg^2+^ absorption in the gastrointestinal tract and Mg^2+^ excretion by the kidneys. Several Mg^2+^ transporters responsible for cellular Mg^2+^ homeostasis have been discovered. On the cell membrane, there are transient receptor potential channels melastatin family member 6 and 7 (TRPM6 and TRPM7), a Na^+^/Mg^2+^ exchanger solute carrier family 41 member 1 (SLC41A1), SLC41A2, cyclin and CBS domain divalent metal cation transport mediator 2 (CNNM2, or ancient conserved domain protein 2—ACDP2), Mg^2+^ transporter 1 (MagT1), and claudin-16 [8,9,10,11,12,13,14]. On the organelles’ membranes, there are mitochondrial RNA splicing 2 protein (MRS2) [15] and SLC41A3 [16] on the mitochondrial membranes and endoplasmic reticulum Mg^2+^ ATPase (ERMA) [17] and AtMGT4 [18] on the endoplasmic reticulum membrane. There are also some exchangers, cotransporters, and antiporters showing capabilities of Mg^2+^ transport [19,20]. Notably, TRPM6 and TRPM7 also have an α-kinase domain to phosphorylate downstream targets. Therefore, they are also called chanzyme, having both channel and enzyme functions. Despite all that has been discovered, we still have a long way to go to understand Mg^2+^ transport and sequestration and the responses of these systems to hypo- or hypermagnesemia.

Disturbed Mg^2+^ homeostasis can be either hypomagnesemia (HypoMg), which is more commonly observed and the focus of this special issue, or hypermagnesemia that is rarer and mostly caused by kidney failure or accidental overdose of Mg^2+^ supplementation. HypoMg can be caused by low Mg^2+^ intake (because starvation, malnutrition, or bad diet habits), genetic disorders [21] (such as mutations of Mg^2+^ transporters [22,23,24]), Mg^2+^ wasting diseases (such as alcohol abuse disorder, diabetes mellitus, celiac disease, and inflammatory bowel disease), and medications that limit Mg^2+^ absorption or escalate Mg^2+^ excretion such as proton pump inhibitors and calcineurin inhibitors that treat cancer. HypoMg has shown deleterious effects on the functions of nerves, brain, heart, bones, and muscles. Symptoms include tremors, muscle tetany, and weakness. Severe HypoMg can cause seizures, cardiac diastolic dysfunction, cardiac arrhythmias, and even death. Mg^2+^ supplementations have been used to treat patients with acute/severe HypoMg by intravenous injection or general populations with mild HypoMg by oral intake.

This special issue focuses on cellular and systemic Mg^2+^ homeostasis regulated by Mg^2+^ transporters in human health and diseases and how Mg^2+^ treatment or supplementation benefits patients. Five manuscripts have been published in this special issue with one Opinion, one research article, one meta-analysis of clinical trials, and two clinical studies.

## 2. An Overview of Published Articles



**Key points of the special issue:**
oMg^2+^ is more than just a cation for ATP.oLow serum Mg^2+^ is an independent risk factor for long-term all-cause mortality in patients with non-ST-elevation myocardial infarction (NSTEMI) and may serve as a relatively inexpensive and fast biomarker for poor outcomes.oLow Mg^2+^ is associated with and may cause increased inflammation and worse procedural outcomes.oMg^2+^ supplementation may suppress oxidative stress, inflammation, and senescence in a variety of diseases.oSome of the effects of low Mg are mediated through regulation of the TRPM6 and TRPM7 kinase activities.



Liu and Dudley presented an Opinion article focused on the correlation between HypoMg, TRPM7, mitochondrial function, and inflammation. The authors proposed that HypoMg is more than just alterations in ion homeostasis. TRPM7 expression is counter-regulated to Mg^2+^ concentration [5,25,26], probably as a compensation. Increased TRPM7 levels result in enhanced TRPM7 kinase signaling. Many of the pathogenic effects of HypoMg on mitochondrial dysfunction, oxidative stress, and inflammation can be attributed to the HypoMg-induced upregulation of TRPM7 kinase function and subsequent signaling pathway, without significant modifications on serum or cellular Mg^2+^ level [5].

The TRPM7 kinase has been reported to phosphorylate other proteins and affect transcription and translation [27,28,29,30,31,32]. TRPM7 kinase inhibition may work well to treat the sequelae of HypoMg and may be especially important for patients who have genetic disorders and cannot maintain a normal serum Mg^2+^ levels even with Mg^2+^ supplementation or who cannot use Mg^2+^ supplementation because of severe side effects such as diarrhea and accidental hypermagnesemia. The remarkable anti-inflammatory and anti-oxidant effects of Mg^2+^ supplementation and possible TRPM7 kinase inhibitors may prove to have a wider application for other diseases characterized by inflammation and oxidative stress with or without symptoms of HypoMg.

Their article discussed HypoMg and its association with a variety of diseases such as diabetes, neurodegeneration diseases, and epilepsy, with a focus on cardiovascular diseases such as heart failure with preserved ejection fraction, arrhythmia, myocardial infarction, diabetic cardiomyopathy, and hypertension. Mg^2+^ transporters were also reviewed concisely with their function, localizations, and specificity. In the end, the authors pointed out a lot of unknowns for future investigation, such as which diseases characterized by inflammation and oxidative stress could respond to Mg^2+^ supplementation and TRPM7 kinase inhibitor therapy, if there are specific cell types mediating the effects of HypoMg, TRPM7, and Mg^2+^ supplementation, and to what extent TRPM7 kinase activity can explain the many effects of HypoMg.

The second contribution is a clinical study. Segev et al. investigated serum Mg^2+^ levels in a large cohort of over 4500 hospitalized patients with non-ST-elevation myocardial infarction (NSTEMI) who were followed for a median of 4.4 years (IQR 2.4–6.6 years). The patients were divided into two groups: low serum Mg^2+^ group (1.6–1.8 mg/dL) and normal/high serum Mg^2+^ group (2.0–2.2 mg/dL). They found that low serum Mg^2+^ is an independent risk factor for long-term, all-cause mortality in NSTEMI patients (~24% higher than patients with normal serum Mg^2+^ levels). This association was still significant after adjustment for demographic and clinical variables.

This article highlights that serum Mg^2+^ levels upon admission may serve as a relatively inexpensive and fast biomarker for poor outcomes and aid in the risk stratification of NSTEMI patients. The paper suggests that HypoMg may be causative of a more debilitated state, and it suggests the possibility that Mg^2+^ correction may improve outcomes in acutely ill HypoMg patients. The low serum Mg^2+^ group was older (mean 72 vs. 67, *p* < 0.001), had more female patients (36% vs. 28%, *p* < 0.001), and had higher rates of having diabetes (59% vs. 29%), hypertension (92% vs. 85%, *p* < 0.001) and atrial fibrillation (6% vs. 5%, *p* = 0.028). Clinical studies have shown that low Mg^2+^ levels are associated with these conditions [33,34,35,36,37,38], although it is unclear if HypoMg results from these conditions or contributes to them. Liu et al. also reported that low serum Mg^2+^ levels contribute to cardiac diastolic dysfunction and heart failure with preserved ejection fraction (HFpEF) [2,3,4]. Therefore, it is possible that low Mg^2+^ causes increased risks of cardiovascular disease. A limitation of this work is that all patients were from a single tertiary medical center, so the results may not be applicable to a wider array of patients.

The third article is a meta-analysis of randomized controlled trials. Hung et al. investigated the effects of intravenous MgSO_4_ infusion on the postoperative quality of recovery assessed with questionnaires of the Quality of Recovery (QoR, QoR-40 and QoR-15). Previously, this group has reported that N-methyl-d-aspartate (NMDA) receptor antagonists such as ketamine and esketamine improve the recovery quality of patients [39]. In this work, Mg^2+^, a NMDA receptor antagonist, was investigated for its potential to alleviate postoperative pain and inflammation. A total 622 patients (46 from USA, 120 from Turkey, 301 from China, and 155 from Korea) from seven randomized controlled trials were analyzed. The benefits of MgSO_4_ infusion elucidated in this work include improved global QoR score, physical comfort, emotional states, and physical independence with less pain. Meanwhile, the intraoperative opioid use and incidence of postoperative nausea and vomiting were reduced by MgSO_4_ infusion. The authors also performed sensitivity analysis that showed the reduction in the intraoperative narcotics consumption and the extubation time were robust findings across the trials.

This study highlights the potential of intravenous MgSO_4_ infusion as a valuable adjunct for multimodal analgesia and enhanced recovery. This work may help direct future studies on MgSO_4_ infusion including the optimization of dosing strategies, administration time, and specific surgical populations. Several limitations are acknowledged, such as high heterogeneity between studies, the short duration of most surgeries studied, unexplored impacts of MgSO_4_ infusion on chronic postsurgical pain, persistent functional impairment, and healthcare resource utilization.

The fourth contribution is an original research article. Laragione et al. explored the biological pathways of Mg^2+^ supplementation on regulating ferroptosis and cell senescence in arthritis. Over 40% of the US population consumes less Mg^2+^ than the required amounts, and rheumatoid arthritis (RA) patients have a diet deficiency in Mg^2+^ [40,41]. With a mouse model of KRN serum-induced arthritis (KSIA), the authors showed that Mg^2+^ supplementation could protect against RA and tissue transcriptomic consequences of the disease. Mg^2+^ supplementation significantly decreased synovial hyperplasia and joint damage in KSIA mice. The KSIA mice had elevated gene expression of the Reactome and Gene Ontology pathways implicated in RA pathogenesis including RHO GTPase, the RUNX1 pathway, oxidative stress-induced senescence, and the senescence-associated secretory phenotype in the synovial tissues. Mg^2+^ supplementation enhanced gene expression involved in anti-oxidative, anti-aging or anti-senescence activity and ferroptosis most significantly, while decreased genes involved in the inflammatory response, ferroptosis, and cell senescence. In summary, oral Mg^2+^ supplementation suppressed the tissue response to oxidative stress and senescence in RA. Therefore, Mg^2+^ supplementation may become a new therapeutic option for RA and other autoimmune and inflammatory diseases.

The fifth entry was another clinical trial concerning the most effective oral delivery state for Mg. Mg^2+^ supplements are often recommended to people. Different forms of Mg^2+^ supplementations have different absorption rates [2,4], but the best oral preparation is unknown. In a double-blind, randomized, cross-over clinical study, Pajuelo et al. compared the effects of oral administration of MAGSHAPE^TM^ microcapsules (Mg-MS, a Mg^2+^ oxide based nutraceutical mix) on plasma Mg^2+^ levels to other widely used Mg^2+^ supplementations including Mg^2+^ oxide (MgO), Mg^2+^ citrate (Mg-C), and Mg^2+^ bisglycinate (Mg-BG). In this work, volunteers were first put on a low-Mg diet (Mg-rich food in Table S1 was excluded from the diet) for 7 days. Then after 8 h of fasting, blood was taken at 0, 1, 4, and 6 h after the oral intake of Mg^2+^ supplementation products. After oral intake of Mg-MS, the plasma Mg^2+^ levels were increased significantly at all tested time points. Nevertheless, the area under the curve (AUC) of the total amount of Mg^2+^ in plasma between the four Mg^2+^ supplementations was not statistically different, suggesting that total Mg^2+^ absorbed was not different between preparation. Mg-MS did show less adverse side effects, however, including reduced intestinal motility and sensations of gastric heaviness that are commonly associated with Mg^2+^ supplementation.

## 3. Future Vision of Mg^2+^ Research and Clinical Uses

Mg^2+^ is not just a cation partner of ATP. Mg^2+^ is inversely related to inflammation, and Mg^2+^ supplementation may reduce inflammation and even postsurgical pain. Understanding of Mg^2+^ homeostasis and Mg^2+^ transporters is still limited, however. The localizations and functions of Mg^2+^ transporters/channels need to be more fully characterized. With this knowledge, specific activators and inhibitors to Mg^2+^ transporters/channels could be developed to better maintain Mg^2+^ homeostasis in diseases.
**What needs further investigation:**oWhich inflammatory diseases will respond to Mg^2+^ supplementation?oTo what extent can TRPM6 and TRPM7 kinase activity explain low Mg^2+^ effects?oWhich cell types are most affected by low Mg^2+^?oWhich Mg^2+^ preparation is best to treat the effects of low Mg^2+^?

Because of its pathogenic effects, low Mg^2+^ may be a biomarker of poor outcomes, and clinical Mg^2+^ tests might be considered routinely for many patients mentioned in this Editorial and any detected HypoMg corrected with Mg^2+^ supplementations to improve clinical outcomes.

Some effects of Mg^2+^ are dependent on its regulation of TRPM6 and TRPM7 kinase function independently from their Mg^2+^ transport functions. The kinases have many phosphorylation targets that modulate protein transcription and translation, amplifying pathological signals of HypoMg. The TRPM7 kinase inhibitor TG100-115 that has shown anti-inflammatory effects [42] is being investigated in a clinical trial as a potential treatment in acute myocardial infarction by reducing inflammation and cardiac damage.

The best way to administer Mg^2+^ is still an active area of research. Oral Mg^2+^ therapy is desirable because of its low cost and long self-life. On the other hand, it is complicated by inadequate absorption or increased gastrointestinal distress. Formulations of Mg^2+^ supplementations with better absorption rates and long-lasting effects may be on the horizon.
